# Effect of Methanolic Extract of Corn Silk on Cisplatin-Induced Nephrotoxicity in Rats

**DOI:** 10.22086/gmj.v0i0.1258

**Published:** 2018-11-29

**Authors:** Nader Tanideh, Fariba Zarifi, Shima Rafiee, Maryam Khastkhodaei, Omid Koohi Hosseinabadi, Firoozeh Tarkesh, Zahra Kherad, Maryam Mojahed Taghi, Mahsa Kamali, Golsa Shekarkhar, Mohamad Jahromi, Farzane Zarifi

**Affiliations:** ^1^Stem Cells Technology Research Center, Shiraz University of Medical Sciences, Shiraz, Iran; ^2^Department of Pharmacology, Shiraz University of Medical Sciences, Shiraz, Iran; ^3^School of Medicine, Shiraz University of Medical Sciences, Shiraz, Iran; ^4^School of Medicine, Tehran University of Medical Sciences, Tehran, Iran; ^5^Department of Anatomical Sciences, School of Medicine, Shiraz University of Medical Sciences, Shiraz, Iran; ^6^Laboratory Animals Center, Shiraz University of Medical Sciences, Shiraz, Iran; ^7^Student Research Committee, Department of Clinical Nutrition, School of Nutrition and Food Sciences, Shiraz University of Medical Sciences, Shiraz, Iran; ^8^Department of Mycology, School of Medicine, Shiraz University of Medical Sciences, Shiraz, Iran; ^9^Department of Pharmacology, School of Medicine, Shiraz University of medical sciences, Shiraz, Iran; ^10^Student Research Committee, Shiraz University of Medical Sciences, Shiraz, Iran; ^11^Pathology Department, Molecular Pathology, Shiraz University of Medical Sciences, Shiraz, Iran; ^12^Clinical Research Unit, Medical Division, Dasman Diabetes Institute, Dasman, Kuwait; ^13^Legal Medicine Research Center, Legal Medicine Organization of Iran, Tehran, Iran

**Keywords:** Corn Silk, Cisplatin, Nephrotoxicity, Lipid peroxidation

## Abstract

**Background:**

Cisplatin is a cytotoxic agent in cancer therapy. Nephrotoxicity is considered as a side effect of cisplatin usage. Using rate models, we studied the possible protective impact of corn-silk (CS) extract against cisplatin-induced nephrotoxicity.

**Materials and Methods:**

Thirty-five experimental rats were divided into five groups (n=7 per each group) as follow: C1: Control received distilled water only; C2: received one dose of cisplatin, and CS: received 300 mg/kg/day of CS. Both CS1 and CS2 received 200 and 300 mg/kg/day of the CS extract orally, individually, for eight consecutive days. CS1 and CS2 received a single dose of cisplatin on the first day only. The specific biochemical markers and histopathological alterations were evaluated.

**Result:**

According to our results, cisplatin administration could have induced severe degeneration in all parts of the nephron tubules and liver. Pre-treatment with CS exhibited a significant decrease in the malondialdehyde (MDA) levels as compared to the values obtained after treatment with cisplatin alone (P<0.01). Moreover, the CS extract with 200 mg dose showed significant (P<0.01) protection against the cisplatin-induced elevation of blood urea nitrogen. Further, the serum levels of alanine transaminase and aspartate transaminase were higher in the cisplatin-treated groups, when compared to the control group (P<0.05). Furthermore, the hepatic function was also improved in cisplatin-treated animals, which were pre-treated with CS.

**Conclusion:**

CS has the potential to attenuate nephrotoxicity and lipid peroxidation induced by cisplatin in rats.

## Introduction

Cisplatin, also named as cis-diamminedichloroplatinum (II), is an anti-neoplastic bifunctional (has properties of two different functional groups) alkylating agent, belonging to the group of platinum anti-tumor compounds. For example, cisplatin is commonly used in chemotherapy of various forms of cancer including head, neck, ovarian, bladder, and testicular cancers [1].

Despite cisplatin beneficial effects, it has considerable nephrotoxic and neurotoxic side effects that often limit its clinical use. For instance, renal proximal tubular toxicity is the major limitation of using cisplatin [2].

Neither prophylactic intensive hydration nor force diuresis has been effective in eliminating cisplatin toxicity [3].

DNA damage and apoptosis induced by cisplatin (oxidative stress) appear to be the mechanisms by which this drug produces renal injury. The oxidative stress resulting from increased free radical generation plays a key role in the pathogenesis of cisplatin-induced nephrotoxicity. Studies have shown that abnormal production of reactive oxygen species (ROS) can cause renal proximal tubule cellular damage and necrosis [4-10].

Corn-silk (CS), stigma/style of Zea mays Linne, is a famous traditional herbal medicine, which has been widely used for the treatment of edema, cystitis, gout, kidney stones, nephritis, and prostatitis [11,12].

CS is known to have a wide range of pharmacological and biological activities [13].

This herb contains proteins, vitamins, carbohydrates, calcium, potassium, manganese and sodium salts, volatile oils, and steroids. The diuretic action of CS is partly due to its significant K content [14-16].

CS is also known to be rich in phenolic compounds such as anthocyanin, p-coumaric acid, vanillic acid, protocatechuic acid, derivatives of hesperidin and quercetin, and bounded hydroxycinnamic acid components composed of p-coumaric and ferulic acid. Phenolic compounds present in CS play an important role in its antioxidant and free radical scavenging capacity [12].

Methanol extracts of CS exerted antioxidant effects against lipid peroxidation [17].

Consumption of CS was also reported to be safe and has no adverse effects for humans [18].

In previous investigations, CS has been shown to have beneficial effects on nephrotoxicity via the amelioration of oxidative injury [19,20].

This study was designed to investigate the possible protective effects of CS against cisplatin-induced nephrotoxicity in rat models.

## Materials and Methods

### Animals

Thirty-five rats aged about 10-12 weeks and with 220 ± 20g mass were obtained from our animal breeding center, Shiraz University of Medical Sciences, Shiraz, Iran.

### Preparation of CS Extract

The CS was collected and authenticated by the department of pharmacology at Shiraz University of Medical Sciences. The plant was dried and powdered at room temperature. The methanol extraction was prepared by soaking the plant powder into 1000ml of 80% methanol.

### Study Groups

The rats were randomly divided into five groups of seven each: C1 as the untreated control group, which received distilled water (1 ml/day) orally for eight consecutive days; group 2, the C2 received a single dose of cisplatin (5 mg/Kg, i.p.) on the first day of study. The CS group received 300 mg/kg/day of the CS extract orally. Groups 4 (CS1) and 5 (CS2) received 200 and 300 mg/kg/day of the CS extract orally, respectively, for eight consecutive days. CS1 and CS2 received a single dose of cisplatin (5mg/Kg, i.p.) post-CS extract dosage only on the first day.

### Sample Collection and Biochemical Measurements

After nine days, all animals were anesthetized with ether; blood samples were prepared by cardiocentesis for measuring the serum levels of alanine transaminase (ALT) and aspartate transaminase (AST), blood urea nitrogen (BUN). The left kidney was removed, homogenized in cold potassium chloride solution (1.5%) to give a 10% homogenate, and used for measuring malondialdehyde (MDA). Lipid peroxidation was measured as the amount of MDA determined by the thiobarbituric acid reactive substance [21].

### Histopathological Evaluations

All the kidneys were fixed by the 10% formaldehyde; then, we prepared 5 µm sections by microtome. All the slides were stained with hematoxylin and eosin. We examined the degree of the presence of congestion and degenerative cellular changes. The level of each pathological manifestation was graded according to the changes involved: none with 0, less than 20 % with 1, 21–40 % with 3, 61–80 % with 4, and greater than 80 % with 5. The sum of all numerical scores in each group was taken as the total histopathological score [22].

### Statistical Analysis

Data are given as mean ± standard error of the mean. The statistically significant differences were assessed using one-way analysis of variance (ANOVA) followed by Tukey’s multiple comparisons, and post hoc test. A P<0.05 was considered as significant level.

## Result

### Effects of CS on Cisplatin-Induced Lipid Peroxidation

**Figure 1:**
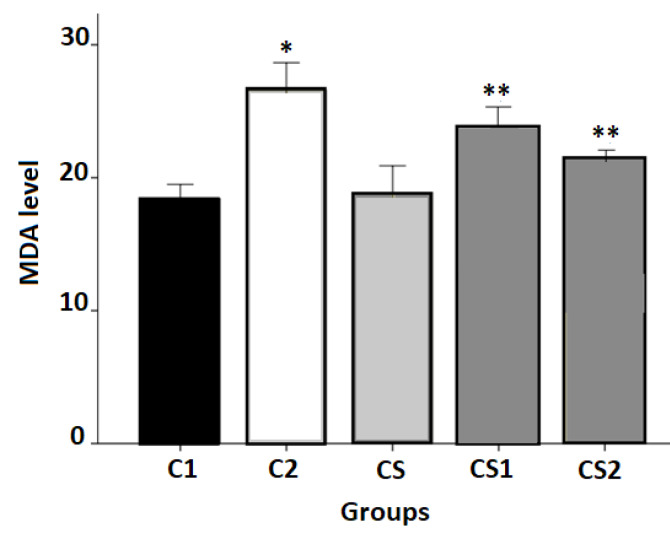
Effects of CS on lipid peroxidation levels (MDA) in rats. Data are presented as mean ±SD. *P<0.01 vs. control group; **P<0.01 vs. cisplatin-treated group

As illustrated in Figure-1, the CS group animals showed no any significant difference compared to the C1 group, whereas the cisplatin-treated groups represented a considerable increase in their levels of MDA (P<0.01). Pre-treatment with CS exhibited a significant decrease in the MDA levels as compared to the values obtained after treatment with cisplatin alone (P<0.01).

### Effects of CS on BUN Level

**Figure 2:**
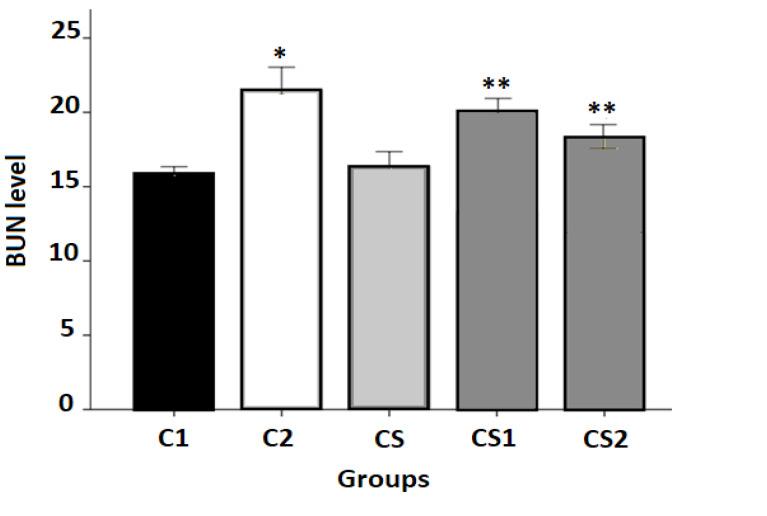
Effects of CS on serum levels of the BUN in rats. Data are presented as mean ± SD. *P<0.01 vs. control group; **P<0.01 vs. cisplatin-treated group

The levels of the BUN were examined in all groups. The cisplatin-treated groups showed a significant increase in the levels of BUN (P<0.01). The CS1 and CS2 animals pre-treated with CS followed by a single dose of cisplatin showed a significant decrease in the level of BUN compared to cisplatin alone (P<0.01, Figure-2).

### Effects of CS on AST and ALT Levels

**Figure 3:**
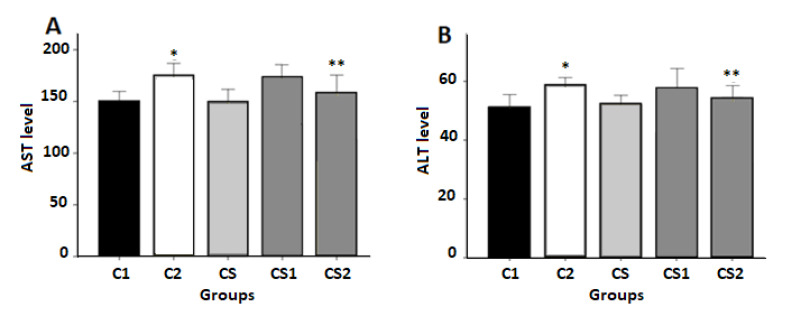
Effects of CS on AST (A) and ALT (B). Data are presented as mean ± SD. *P<0.01 vs. control group; **P<0.01 vs. cisplatin-treat

AST and ALT levels were higher in the cisplatin-treated groups when compared to the control group. In contrast, the CS2 rats revealed a significant decrease in the level of the same parameters in comparison to C2 (P<0.05). However, the hepatic function was improved in cisplatin-treated animals, which were pre-treated with CS (Figure-3).

### Effect of CS on Histopathological Changes

**Figure 4:**
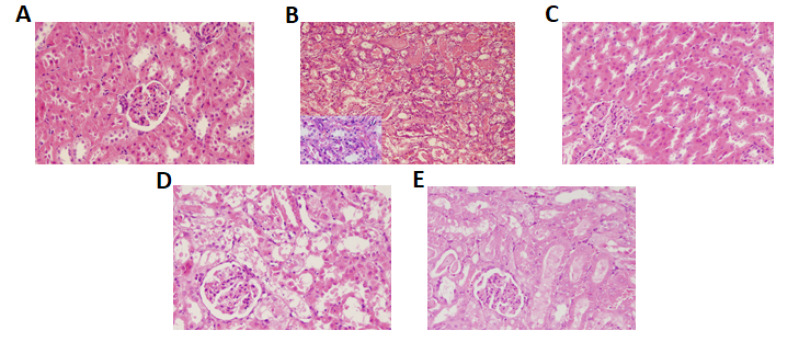
Histological sections of the kidney (H&E staining, ×200) in experimental rats. A: Group C1 showing adequate tissue with no specific pathologic change; B: Group C2 showing extensive tubular necrosis and desquamation as well as signs of regeneration magnified in lower left of the image; C: Group CS showing minimal damage, focal loss of brush borders, and very few hyaline casts; D: Group CS1showing moderate tubular necrosis and cast formation admixed with normal tubules; E: Group CS2 showing minimal tubular necrosis.

Histological findings of the kidney from various treatment groups are presented in Figure-4. Treatment with cisplatin caused extensive tubular necrosis and desquamation. The pre-treatment with CS decreased the tubular necrosis compared to cisplatin alone.

## Discussion

Cisplatin is considered as one of the active cytotoxic agents in the treatment of cancer. However, nephrotoxicity, hepatotoxicity, and neurotoxicity are considered as cisplatin’s side effects [23]. Previous researches have revealed that cisplatin induces nephrotoxicity via free radicals and ROS due to the glutathione level and antioxidant enzyme depletion [24,25]. In recent years, there has been an attempt to use extracts from medicinal plants with antioxidant properties to treat different diseases [19,26]. Based on previous studies, CS is one of the herbs with antioxidant and free-radical scavenger activities that can be used in renal damage and oxidative injury [11,19]. In the current study, the effect of CS on cisplatin-induced nephrotoxicity was investigated using experimental rat models.

Suzuki et al. investigated the effects of CS on diabetic nephropathy induced by streptozotocin (STZ). Based on their results, the increase in creatinine clearance in the administered group (STZ+ CS) was suggestively inhibited compared with the non-treated group (STZ). Such a trend was also observed in urinary albumin excretion. The latter results showed that the aquatic extract of CS prevented the glomerular hyperfiltration. Accordingly, the water extract of CS suppressed the development of diabetic glomerular sclerosis in STZ-induced diabetic rat [27].Sepehri et al. in their experimental study evaluated the effect of CS against gentamicin (GM)-induced nephrotoxicity. They used different doses of CS (200, 300, 400 and 500 mg/kg). As per their results, plasma creatinine and urea levels significantly increased in the GM group. CS management (200 and 300 mg/kg) with GM injection significantly reduced the serum creatinine, but not urea levels compared with the GM group. Acute tubular necrosis hyaline casts in the tubular lumen, interstitial nephritis, and glomerular variations were observed in the GM group, using histopathological techniques. Notably, co-treatment of CS with GM considerably decreased the interstitial nephritis. Furthermore, high doses of CS caused hyaline cast formation, apoptosis, congestion, and swelling of the renal tubules. Consequently, CS might improve nephropathy during prolonged therapeutic use of GM and related aminoglycosides [19].In the present study, we demonstrated that daily CS administration significantly improved the cisplatin toxicity to the kidney and liver, as confirmed by biochemical and microscopic examination. Cisplatin administration showed a significant increase in the urea levels compared to normal rats, which indicated renal failure.Based on our results, cisplatin administration caused severe degeneration in the glomeruli and both proximal and distal tubules. Functional nephrotoxicity indices, such as the BUN, were increased in the cisplatin-injected animals compared to the control group. Furthermore, cisplatin administration caused severe damage in the liver, as assessed microscopically. The CS extract with 200 mg dose protected the elevation of the cisplatin-induced BUN. The histopathological evaluation also showed that pre-treatment with CS caused a significant reduction in the tubular necrosis compared to cisplatin alone which is in the same line with Ingale et al. report [4].

Although the exact mechanism of nephrotoxicity induced by cisplatin is not well understood [28], previous reports have suggested that the free radicals and ROS are involved due to the depletion of glutathione concentration and antioxidant enzyme activities in the kidney [29,30]. The above observations support the mechanism of nephrotoxicity induced by cisplatin in animals, which is partially related to the depletion of the renal antioxidant system. Treatment of cisplatin-induced nephrotoxicity with CS could significantly prevent the reduction of these renal antioxidant systems. The quantitative stereological evaluation was not performed, which was one of the limitations of this study.

## Conclusion

CS has the potential to attenuate nephrotoxicity and lipid peroxidation induced by cisplatin in rats.

## Conflict of Interest

All authors declare there is no any conflict of interest in presenting the current work.
